# Diagnostic performance of blood-based liquid biopsies in hepatocellular carcinoma: A protocol for systematic review and meta-analysis

**DOI:** 10.1097/MD.0000000000022594

**Published:** 2020-10-09

**Authors:** Ting Zhang, Minggang Yin, Lizhen Wang, Wenzhai Cao, Sen Zhong

**Affiliations:** a Department of Medicine, Sichuan Vocational College of Health and Rehabilitation; b Department of Cardiology, Zigong First People's Hospital, Zigong; c School of Clinical Medicine, Chengdu University of Traditional Chinese Medicine, Chengdu, Sichuan, PR China.

**Keywords:** circulating tumor DNA, circulating tumor cells, diagnosis, exosomes, Hepatocellular carcinoma, liquid biopsy

## Abstract

**Background:**

Hepatocellular carcinoma (HCC) is an aggressive cancer associated with poor prognosis. Early diagnosis is crucial to improve its prognosis. Blood-based liquid biopsies are promising methods in detecting HCC. However, their accuracies have not been systematically assessed, so it is essential to conduct a meta-analysis to evaluate the diagnostic performance of blood-based liquid biopsies in detecting HCC.

**Methods:**

We will search PubMed, EMBASE, Cochrane Library, Web of Science, Medline, China National Knowledge Infrastructure(CNKI) for the relevant studies that assessed the diagnostic performance of blood-based liquid biopsies including circulating tumor cells(CTCs), circulating tumor DNA(ctDNA), and exosomes(EVs) in HCC patients from inception to September 2020. Two researchers will independently extract the data and use Quality Assessment of Diagnostic Accuracy Studies-2 (QUADAS-2) to evaluate the quality of included literature. We will also conduct the pool diagnostic value, heterogeneity across studies and reporting bias. All the statistical analysis will be conducted by Stata V.15.0 and Meta-disc V.1.4.

**Results:**

This review will evaluate the pooled diagnostic value of blood-based liquid biopsies in HCC.

**Conclusion:**

This review will summarize the current published evidence of blood-based liquid biopsies in diagnosing HCC, which may provide a great opportunity for promotion and application of them.

**Open Science Framework(OSF) registration number:**

September 3, 2020. https://osf.io/9n4yz.

## 1 Introduction

Hepatocellular carcinoma (HCC), characterized by significant morbidity and mortality, is the third common cancer and second dominating cause of tumor-related death worldwide.^[1]^ Early diagnosis of HCC could greatly improve the prognosis of the disease through the available treatment, such as surgery, liver transplant. However, due to delayed diagnosis, more than 70% of HCC patients are not suitable for surgery.^[2]^ Alpha-fetoprotein (AFP), the most common clinical biomarker, seems insufficient value in early diagnosis of HCC with a low sensitivity of 63% and a specificity of 84%.^[3]^ Imaging methods, including CT, MRI, and CEUS have improved the sensitivity from 66% to 82% and the specificity up to 90%, but the diagnostic efficiency of HCC is merely applicable to nodules larger than 1 cm diameter.^[4]^ Therefore, more rapid and robust methods are crucial for early HCC diagnosis.

Liquid biopsies, which analyze the sample released into the bloodstream or other body fluids from solid tumors, could represent the evolving landscape of cancer in real-time and might be promising cancer biomakers.^[5]^ Blood-based liquid biopsies detect tumor-associated components in the blood including circulating tumor cells (CTCs), circulating tumor DNA(ctDNA), and exosomes(EVs).^[6,7]^ These methods are feasible, repeatable, and mini-invasive alternative to tissue biopsy. In recent years, the diagnostic performance of blood-based liquid biopsies in HCC diagnosis has been widely studied.^[8–10]^ However, these studies evaluated the sensitivities and specificities of a single liquid biopsy method. Besides, there were considerable discrepancies from inconsistent subjects, diverse detection techniques, and inconsistent results. Therefore, the diagnostic value of blood-based liquid biopsies still unclear. In our study, we will perform a meta-analysis to evaluate the diagnostic efficiency of these liquid biopsies in detecting HCC.

## 2 Methods

### 2.1 Study registration

The protocol of the systematic review has been registered. Registration: OSF Preregisration. September 3, 2020, https://osf.io/9n4yz. It has been reported following the guideline of Preferred Reporting Items for Systematic Reviews and Meta-Analysis Protocol statement.^[11]^

### 2.2 Inclusion criteria for study selection

#### 2.2.1 Type of studies

This review will include original studies that evaluate the diagnostic value of blood-based liquid biopsies (including CTCs, ctDNA, or EVs) in HCC.

#### 2.2.2 Type of participants

Patients who were diagnosed with HCC via histopathology will be included in the review, regardless gender, age, HCC stage, and severity of HCC.

#### 2.2.3 Type of index test

Index test: blood-based liquid biopsies (including CTCs, ctDNA, or EVs) were used in the detection of HCC. Whereas case reports, reviews, cell, or animal studies will be excluded.

#### 2.2.4 Outcome measurements

The Pooled SEN, SPE, PLR, NLR, DOR, AUC, and their 95%*CI*.

### 2.3 Data sources and search strategy

We will search PubMed, EMBASE, Cochrane Library, Web of Science, Medline, China National Knowledge Infrastructure (CNKI) for the relevant studies that assessed the diagnostic performance of blood-based liquid biopsies including circulating tumor cells(CTCs), circulating tumor DNA(ctDNA), and exosomes (EVs) in HCC patients from inception to September 2020. The search strategy of Medline was demonstrated in Table 1. Other electronic databases will be used the similar retrieval strategies.

**Table 1:**
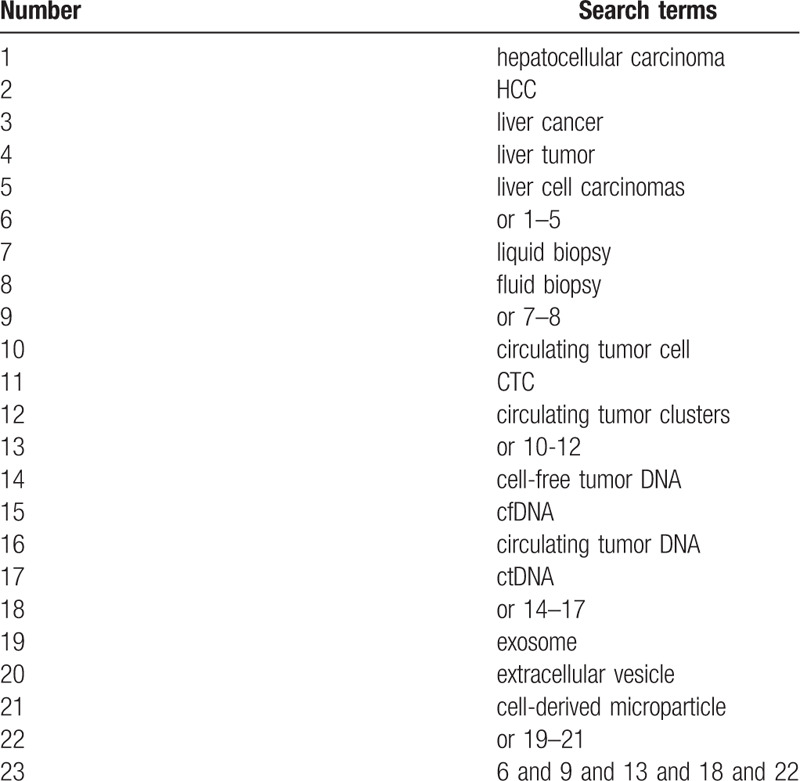
Search strategy applied in MEDLINE database.

### 2.4 Data collection and analysis

#### 2.4.1 Study selection

Two reviewers (TZ and YM) will filtrate the titles and abstracts independently. Then, the full text of potential articles will be appraised for further selection according to the inclusion and exclusion criteria. Any disagreements will be adjudicated by the third researcher (CZ).

#### 2.4.2 Data extraction

Two reviewers (TZ and YM) will independently use a standardized form to extract the data. If there are missing or ambiguous information, we will contact the authors for confirmation. Any disagreements in this process will be resolved through discussion and the standardized forms will be checked by ZS. The data extracted from the studies are first author, publication year, regions, liquid biopsy method, assay indicators, sample size, sample types, cancer stage, control group, data needed for diagnostic meta-analysis (sensibility and specificity data).

### 2.5 Quality assessment

The quality of the included studies will be judged by Quality Assessment of Diagnostic Accuracy Studies-2 (QUADAS-2) criteria in RevMan 5.3 software.^[12]^ The quality assessment consists of 4 key domains: patient selection, index test, reference standard and flow and timing, evaluated the risk of bias and concerns about clinic applicability of all included publications. Two reviewers (TZ and YM) will independently and blindly evaluate the research. Any discrepancies will be solved by consensus.

### 2.6 Statistical analysis

We will compute the pooled SEN, SPE, PLR, NLR, DOR, and their 95% CI. Furthermore, the pooled diagnostic efficiency of blood-based liquid biopsies through the SROC and AUC will be tested. The Spearman correlation coefficient between the logit of sensitivity and logit of 1-specificity will be used to assess the threshold effect, and a *P* value < .05 indicates a significant threshold effect. Heterogeneity caused by non-threshold effect will be evaluated by means of the Cochran Q test and the inconsistency index (*I*?) measurement.^[13]^ Heterogeneity will be deemed significant with *P* < .1 or *I*^2^ > 50%, and a random-effects model will be used. All the statistical analysis will be conducted by Stata V.15.0 and meta-disc V.1.4. *P* values less than .05 will be considered to be statistically significant.

### 2.7 Subgroup analysis

To further study the underlying heterogeneity, subgroup analyses will be conducted as follow:

1. The type of blood-based liquid biopsies (all liquid biopsy methods, CTCs, ctDNA, and EVs).
2. The stage of HCC.
3. The type of samples (whole blood, plasma, serum).

### 2.8 Sensitivity analysis

This review will conduct sensitivity analysis to detect the stability of research results. If certain studies substantially alter the pooled RR in the result of meta-analysis, they will be excluded.

### 2.9 Reporting bias

Publication bias will be tested via funnel plots as well as associated regression tests.^[14,15]^

### 2.10 Ethics and dissemination

This review will extract data from published studies, so examination and agreement by the ethics committee are not required in this study. It will be published in a relevant peer reviewed journal.

## 3 Discussion

Hitherto, histopathology still be regarded as a gold standard for cancer detection. Nevertheless, apart from invasive and non-routine nature, biopsies merely reflect a limited snapshot of the tissue, failing to reflect the cancer landscape and progression. Therefore, to facilitate the evolvement of “precision medicine”, it is urgent to search diagnostic tools that provide timely and accurate information for cancer patients.

In recent decades, liquid biopsies have shown unique advantages as well as distinguished value in cancer diagnosis. Blood-based liquid biopsies such as CTCs, ctDNA, and EVs are attractive targets of HCC biomarkers development.^[16]^ CTCs, cancer cells shed from the tumor tissue in the bloodstream, could be detected at the early stages of HCC.^[17]^ ctDNA mutational, which uniquely occurs early in tumorigenesis, has been investigated for the detection and diagnosis of HCC.^[18]^ EVs, which affect cell-cell communication and are steady present in the blood, act as prospective biomarkers for HCC by containing genetic materials.^[19]^ However despite the range of liquid biopsies currently under study, a systematic review and meta-analysis are needed to compile and synthesize the available data and compare the diagnostic efficacy among different liquid biopsies methods.

This is the first meta-analysis to comprehensively study and generalize the evidence on the potential of blood-based liquid biopsies in diagnosis of HCC. The results of the review will provide clinical evidence and represent a possibility as well as future direction of HCC diagnosis.

## Author contributions

**Conceptualization:** Wenzhai Cao, Sen Zhong.

**Data curation:** Ting Zhang, Minggang Yin.

**Formal analysis:** Ting Zhang, Minggang Yin.

**Methodology:** Ting Zhang, Wenzhai Cao, Sen Zhong.

**Project administration:** Wenzhai Cao, Sen Zhong.

**Resources:** Ting Zhang, Minggang Yin, Lizhen Wang.

**Software:** Ting Zhang, Lizhen Wang.

**Supervision:** Wenzhai Cao, Sen Zhong.

**Writing – original draft:** Ting Zhang.

**Writing – review & editing:** Wenzhai Cao.

## Abbreviations

Abbreviations: AFP = alpha-fetoprotein, AUC = area under the curve, CI = confidence interval, CNKI = China National Knowledge Infrastructure, CTCs = circulating tumor cells, ctDNA = circulating tumor DNA, DOR = diagnosis odds ratio, EVs = exosomes, HCC = hepatocellular carcinoma, NLR = Negative likelihood ratio, PLR = positive likelihood ratio, QUADAS-2 = Quality Assessment of Diagnostic Accuracy Studies-2, SROC = summary receiver operating characteristic.
